# Short-term HIIT impacts HDL function differently in lean, obese, and diabetic subjects

**DOI:** 10.3389/fphys.2024.1423989

**Published:** 2024-08-21

**Authors:** Lin Zhu, Julia An, Thao Luu, Sara M. Reyna, Puntip Tantiwong, Apiradee Sriwijitkamol, Nicolas Musi, John M. Stafford

**Affiliations:** ^1^ Tennessee Valley Health System, Veterans Affairs, Nashville, TN, United States; ^2^ Department of Medicine, Division of Diabetes, Endocrinology and Metabolism, Vanderbilt University Medical Center, Nashville, TN, United States; ^3^ Diabetes Division, University of Texas Health Science Center at San Antonio, San Antonio, TX, United States; ^4^ Division of Human Genetics, School of Medicine, The University of Texas at Rio Grande Valley, Edinburg, TX, United States; ^5^ Department of Medicine, Cedars-Sinai Medical Center, Los Angeles, CA, United States; ^6^ Department of Molecular Physiology and Biophysics, Vanderbilt University, Nashville, TN, United States

**Keywords:** HIIT exercise training, hyperlipidemia, HDL function, obese, antioxidant capacity, cholesterol efflux

## Abstract

**Introduction:**

High density lipoproteins (HDL) exert cardiovascular protection in part through their antioxidant capacity and cholesterol efflux function. Effects of exercise training on HDL function are yet to be well established, while impact on triacylglycerol (TG)-lowering has been often reported. We previously showed that a short-term high-intensity interval training (HIIT) program improves insulin sensitivity but does not inhibit inflammatory pathways in immune cells in insulin-resistant subjects. The purpose of this study is to evaluate HDL function along with changes of lipoproteins after the short-term HIIT program in lean, obese nondiabetic, and obese type 2 diabetic (T2DM) subjects.

**Methods:**

All individuals underwent a supervised 15-day program of alternative HIIT for 40 minutes per day. VO_2peak_ was determined before and after this training program. A pre-training fasting blood sample was collected, and the post-training fasting blood sample collection was performed 36 hours after the last exercise session.

**Results:**

Blood lipid profile and HDL function were analyzed before and after the HIIT program. Along with improved blood lipid profiles in obese and T2DM subjects, the HIIT program affected circulating apolipoprotein amounts differently. The HIIT program increased HDL-cholesterol levels and improved the cholesterol efflux capacity only in lean subjects. Furthermore, the HIIT program improved the antioxidant capacity of HDL in all subjects. Data from multiple logistic regression analysis showed that changes in HDL antioxidant capacity were inversely associated with changes in atherogenic lipids and changes in HDL-TG content.

**Discussion:**

We show that a short-term HIIT program improves aspects of HDL function depending on metabolic contexts, which correlates with improvements in blood lipid profile. Our results demonstrate that TG content in HDL particles may play a negative role in the anti-atherogenic function of HDL.

## 1 Introduction

Heart disease is the leading cause of death worldwide, and coronary artery disease (CAD) is the most common type of heart disease (Tsao et al, 2023). Atherosclerosis is the fundamental cause of CAD, and insulin resistance, dyslipidemia, and chronic inflammation are risk factors that drive the initiation and progression of atherosclerosis. Lifestyles, including daily physical activity, diet, and smoking independently regulate the metabolic risk factors for atherosclerosis. Evidence from cross-sectional studies shows that CAD is delayed in athletes compared to general populations, and CAD risk is lower in physically active than in sedentary individuals (Lee et al., 2012). However, the mechanisms for CAD protective effects of exercise training are not yet well established.

Dyslipidemia comprises multiple aspects, including hyperlipidemia, high lipid content in atherogenic lipoprotein particles (i.e., VLDL and LDL), and low HDL-cholesterol (HDL-C) levels. Regular exercise training improves hyperglycemia and hyperlipidemia by increasing energy expenditure and insulin sensitivity with or without changing body weight (Kraus et al, 2002; Stolinski et al, 2008). The improvement in hyperlipidemia severity is primarily attributed to decreases in lipid contents in VLDL and LDL particles because circulating cholesterol and triacylglycerol (TG) are mainly distributed in those particles compared to HDL particles in humans. The effects of exercise training on HDL-C concentrations reported in prospective studies are controversial. The variability observed may be related to the subjects’ age and baseline lipid status, the amount of fat lost with exercise, and the volume of exercise training (Franczyk et al, 2023).

Aggressively increasing HDL-C concentrations has been an appealing target to reduce CAD events since a Framingham study reported low HDL-C concentrations as a CAD risk (Castelli, 1988). However, clinical trials have not shown promising effects on reducing CAD events by pharmaceutical approaches to increase HDL-C, shifting the focus on HDL function regarding CAD risk. LDL particles retained in the subendothelial space during hyperlipidemia are prone to oxidation (Linton et al, 2023). In response to the oxidized LDL, endothelial cells secrete cytokines to recruit inflammatory cells to the arterial wall to promote atherosclerosis progression. Apolipoproteins associated with HDL particles protect LDL from oxidation to prevent endothelial cells from inflammation (Linton et al., 2023). In addition, HDL particles accept cholesterols transported from the arterial wall and deliver the cholesterols to the liver to favor their secretion (Linton et al., 2023).

Studies have indicated that exercise training at high intensities or training for long periods is likely to increase HDL-C concentrations (Kraus et al., 2002; Valimaki et al, 2016; Wood et al, 2023). The impact of exercise training on HDL function is not yet well studied. High-intensity interval training (HIIT) involves exercise performed at more than 80% VO_2peak_ and interspersed by rest or lower-intensity exercise. HIIT programs often improve cardiometabolic health in less time when measured against high volume continuous exercise (Gillen and Gibala, 2014). In the current study, we examine the impact of an HIIT program on lipid profiles and HDL function in lean, obese, and type 2 diabetic (T2DM) subjects. We show that a short-term HIIT program improves the antioxidant capacity of HDL in all subjects from different metabolic groups and increases the cholesterol efflux capacity in lean subjects.

## 2 Methods and materials

### 2.1 Exercise participants and training

Participants and the exercise training regime have been reported previously (Reyna et al, 2013). Due to the limited availability of fasting blood samples, six samples were randomly used from each group in the current study. All subjects were sedentary and had stable body weight for 3 months before the study. The T2DM subjects were diagnosed no longer than 2 months, and one T2DM subject took a sulfonylurea, which was stopped 2 days before any study procedure. The anthropometric and geographic data for subjects used in the current analysis are shown in Supplement Table 1. Subjects undertook a supervised 15-day program of alternative HIIT for 40 min per day. VO_2peak_ was determined using a cycle ergometer before and after the HIIT program. The HIIT consisted of four identical 10-min periods, which included 8 min of cycle ergometer exercise at 70% of VO_2peak_ followed by 2 min at 90% of VO_2peak_. Each of these 10-min sets was followed by 2 min of complete rest (Reyna et al., 2013). The study was approved by the Institute Review Board of the University of Texas Health Science Center at San Antonio and all participants gave written consent (Reyna et al., 2013). Plasma used in the current analysis was from blood samples collected before the insulin clamp study pre- and post-exercise training. To minimize the influence of acute physical exercise on blood lipid and other laboratory test results (Christou et al, 2017), the post-exercise training insulin clamp and blood sample collection were performed 36 h after the last exercise session (Reyna et al., 2013).

### 2.2 Blood lipid analysis

VLDL, LDL, and HDL particles were separated from plasma using fast performance liquid chromatography (FPLC) (Zhu et al, 2016; Zhu et al, 2018b). Cholesterol and triglycerides in serum and FPLC fractions were determined by enzymatic colorimetric assays with Cholesterol Reagent and Triglycerides GPO Reagent Kits from Infinity (Zhu et al., 2016; Zhu et al., 2018b).

### 2.3 Immunoblotting with plasma samples

For serum proteins, 2 μL (μL) of serum for each sample were denatured in loading buffer (Invitrogen) containing reducing buffer (Invitrogen) and phospholipase and protease inhibitors (Sigma). Serum proteins were separated with gel electrophoresis and transferred to nitrocellulose membranes (Zhu et al., 2016; Mueller et al, 2018). Membranes were incubated with primary antibody (1:1,000) at 4°C overnight, and with secondary antibody (1:20,000) at room temperature for 1 h. ApoB antibody (LS-C20729) was from Lifespan Biosciences; apoA1 antibody (K23500R) was from Biodesign Meridian LifeScience; apoE (ab150032) antibody was from Amcam; apoC2 (PA1-16813) and apoC3 (PA5-78802) antibodies were from Invitrogen. IRDye 800CW goat anti-rabbit IgG was from LI-COR Biosciences. Blot densities were analyzed with ImageJ.

### 2.4 Antioxidant assays

The antioxidant capacity of HDL particles was first assayed by examining their ability to protect LDL from oxidation using kinetics of LDL oxidation in the presence of copper with the protocol we reported previously (Zhu et al, 2018a). ApoB-free serum was prepared as we reported before (Zhu et al., 2018b). One μg of apoB-free serum purified from each sample was added in 300 μL of reaction mix containing 30 μg of LDL (Alfa Aesar) and 20 μM CuSO4 in DPBS. Human HDL particles from Cell Biolabs (Cat# STA-243) were used for the dose-response curve and as positive control, the reaction without HDL was used as blank. Reaction solutions were incubated at 37^o^C for 3 h in a plate reader. The absorbance readings at 234 nm (A234) were taken every minute to measure the formation of conjugated dienes.

The antioxidant capacity of HDL particles was also investigated by quantifying the ability to reduce copper II to copper I using an OxiSelect Total Antioxidant Capacity Assay Kit (Cell Biolabs, Cat# STA-360) following the manufacturer’s introduction. The total antioxidant power of each sample was converted and presented as Copper Reducing Equivalence (CRE) according to Trachootham et al (2008).

Plasma paraoxonase activity was examined using an EnzCheck Paraoxonase Assay Kit from Invitrogen (Cat# E33702) according to Jarvik et al (2003).

### 2.5 Cholesterol efflux assays

The cholesterol efflux capacity of HDL was assayed with apoB-free serum from each sample using a protocol we had reported previously (Zhu et al., 2018a; Zhu et al., 2018b). Briefly, human THP-1 monocytes were differentiated into macrophages after culturing with DMEM containing phorbol myristate acetate (PMA) for 48 h. Cells were then incubated with ^3^H-acylated-LDL particles for 48 h. These labeled foam cells were washed twice and equilibrated in DMEM with 0.2% BSA overnight. The following day, a set of cells were collected and lysed to determine the baseline ^3^H-radioactivities in cells. The remaining cells were treated with 1% of the initial serum level of pooled plasma of each group for 4 h. Cells treated with DMEM with 0.2% BSA were used as negative controls, and cells treated with apoA1 (50 mg/mL, Meridian) as positive controls. ^3^H-radioactivities in media and baseline cells were determined using liquid scintillation counting and used for cholesterol efflux calculation. The efflux rates were calculated as the portion of ^3^H-radioactivities in media to the ^3^H-radioactivities in the baseline cells.

### 2.6 Statistical analysis

Data are shown with pre- and post-exercise training results for individual subjects in each metabolic group. Statistical analysis was performed with one-way or two-way ANOVA with Tukey’s multiple comparison test as indicated in each figure legend. *p*-values <0.05 were considered statistically significant.

## 3 Results

### 3.1 The short-term HIIT modified apolipoproteins differently in lean, obese, and T2DM subjects.

To understand the beneficial effects of exercise training regarding CAD risk, we performed a 15-day high intensity interval training (HIIT) program with lean, obese, and T2DM subjects. In addition to the physiological parameters of participants previously reported (Reyna et al., 2013), we show that the short-term HIIT reduced fasting plasma cholesterol levels in T2DM subjects and fasting TG levels in subjects from obese and T2DM groups (Supplemental Table 1).

**FIGURE 1:**
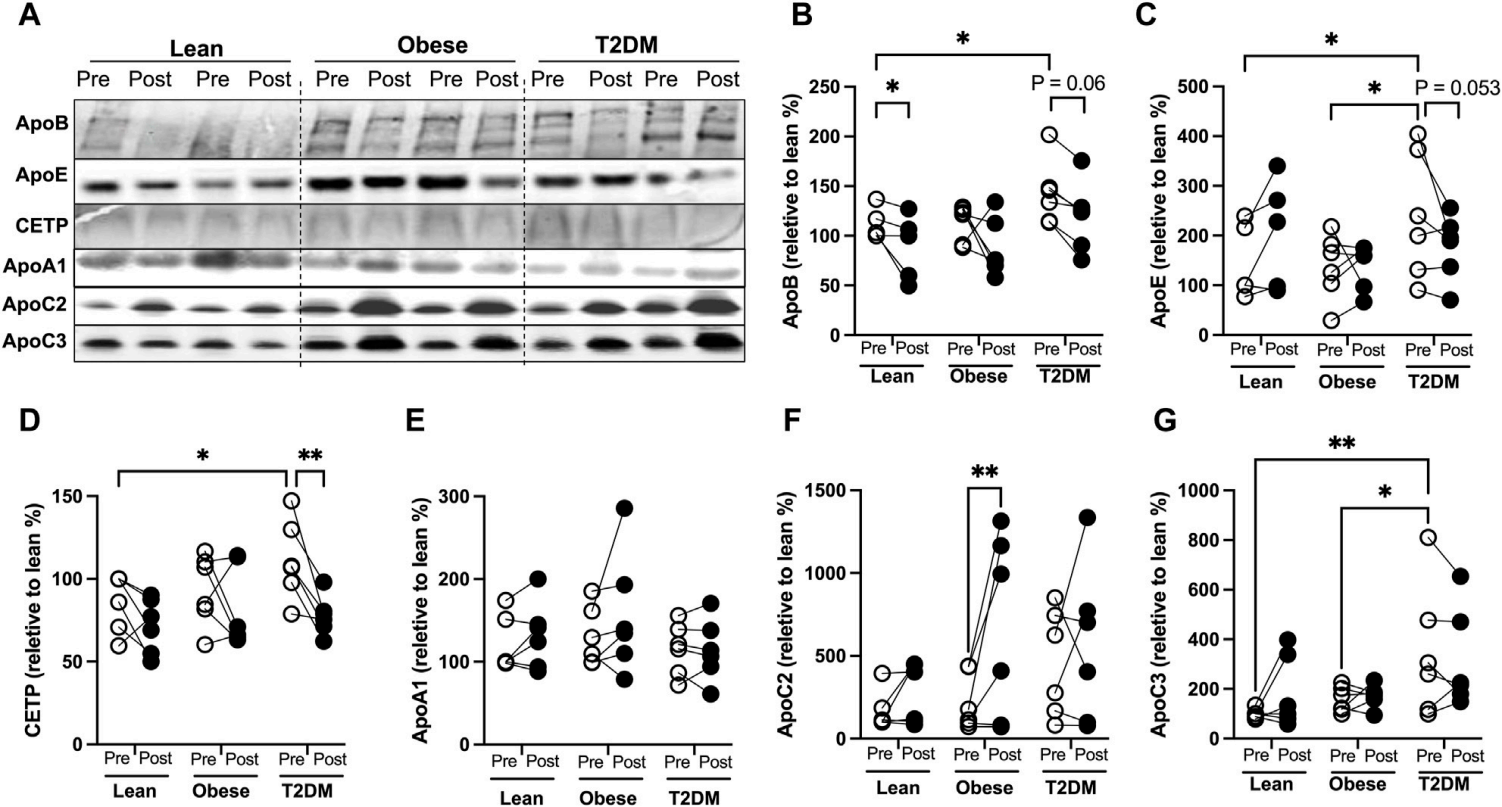
Changes of circulating apolipoproteins by the HIIT program in lean, obese, and T2DM subjects. **(A)** Representative Western blots (WB) for apolipoproteins in plasma pre- and post-exercise training from each subject in lean, obese, and T2DM subjects. All the samples were divided into three groups for WB with each group composed of two leans, two obese, and two T2DM subjects. **(B–G)** Blots was quantified as changes relative to pre-exercise training of lean subjects (first lane) on each WB gel. Open circles represent pre-exercise training expression and dark filled circles represent post-exercise training expression of proteins. Statistical analysis were performed with 2-way ANOVA with Tukey’s multiply comparison analysis, *p* < 0.05 were considered statistically significant.

To better understand the changes in plasma lipids modified by the HIIT, we examined protein amounts of circulating apolipoproteins that are known to regulate lipid delivery and lipolysis. ApoB is the structural protein of atherogenic particles of VLDL and LDL, with a ratio of 1:1 for each particle. The HIIT reduced plasma apoB proteins in lean (*p* < 0.05) participants but not significantly in T2DM (*p* = 0.06) or in obese participants (Figures 1A, B). Plasma apoE protein concentrations have been reported to be higher in T2DM patients than in healthy controls (Franczyk et al., 2023), and plasma cholesterol ester transport protein (CETP) amounts are reportedly higher in hyperlipidemia individuals and considered atherogenic (McPherson et al, 1991). In the current study, plasma apoE protein amounts were not different between lean and obese participants but were increased in T2DM participants compared to lean (*p* < 0.05) and obese participants (*p* < 0.05, Figure 1C). Plasma CETP amounts were higher in T2DM subjects than in lean and obese participants (Figures 1A, D). The HIIT showed a trend to reduce apoE (*p* = 0.053) and reduced CETP proteins (*p* < 0.01) in T2DM participants (Figure 1C).

ApoA1 is the structural protein for HDL particles. Plasma apoA1 protein amounts were not significantly different between metabolic groups or changed by the HIIT in any group (Figure 1E). The human apoCs play an important role in regulating lipoprotein lipase (LPL) activity. ApoC2 is an activator for LPL activity, and decreased apoC2 activities are inversely associated with hypertriglyceridemia in humans (Jong et al, 1999). ApoC3 is an inhibitor for LPL-mediated lipolysis, and increases in apoC3 activity contribute to the development of hyperglyceridemia (Jong et al., 1999). We observed that plasma apoC2 amounts were not different between groups, and the short-term HIIT significantly increased apoC2 only in obese subjects (*p* < 0.01, Figure 1F). Plasma apoC3 amounts were not different between lean and obese subjects and were higher in T2DM than in lean and obese participants (Figures 1A, G). The HIIT program did not significantly change plasma apoC3 in either lean, obese, or T2DM participants (Figure 1G). Interestingly, multiple logistic regression analysis showed that changes in apoC2 were inversely associated with changes in atherogenic factors, including fasting cholesterol, apoB, and insulin levels, while changes in plasma apoC3 proteins positively correlated with changes in fasting cholesterol, TG, and CETP (Supplemental Figure 1).

### 3.2 The short-term HIIT improved different aspects of lipid distribution in plasma lipoproteins in obese and T2DM subjects

**FIGURE 2:**
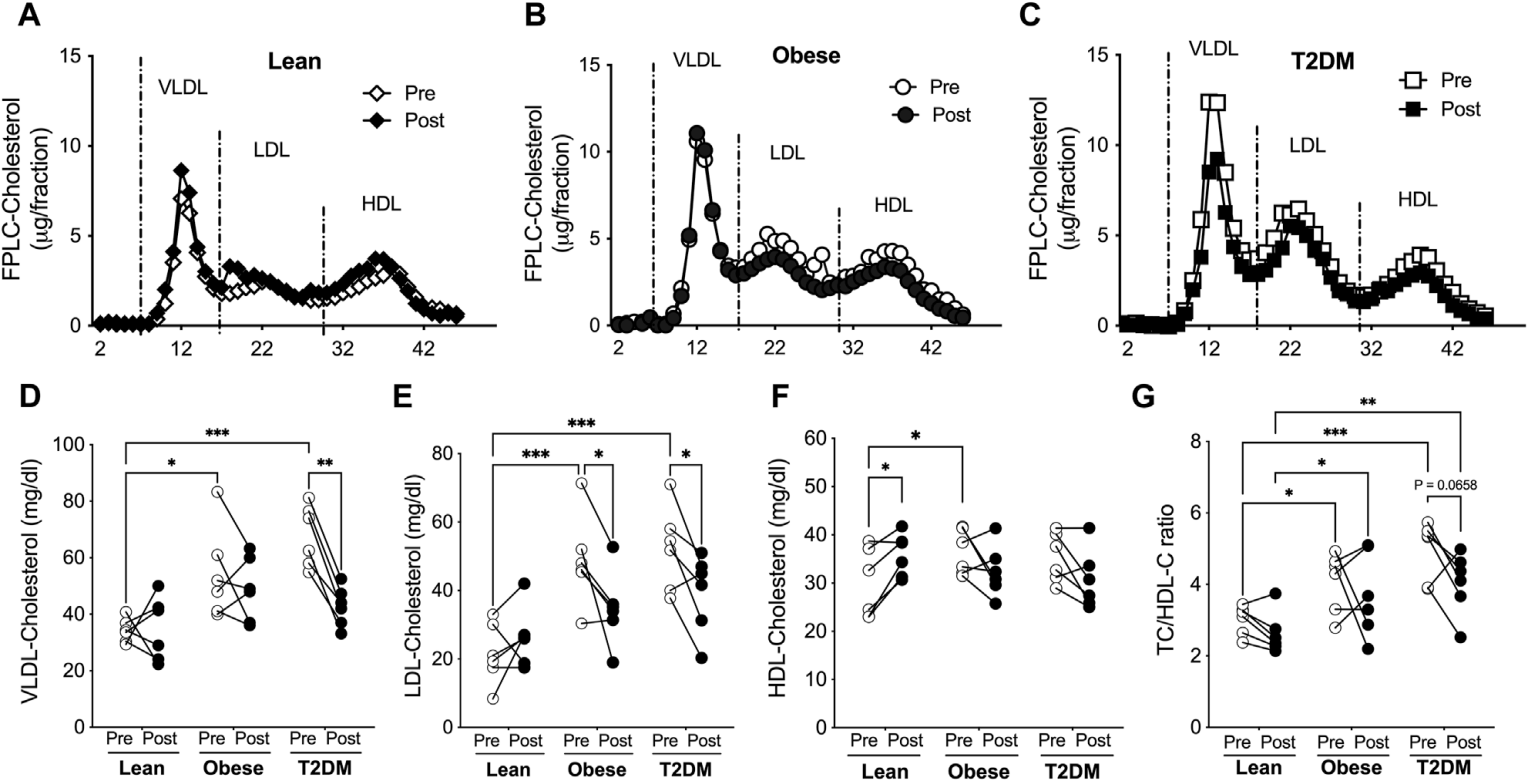
Short-term HIIT modified plasma cholesterol distribution in lipoproteins differently in lean, obese, and T2DM subjects. Plasma lipoproteins were saperated with FPLC and lipoprotein cholesterol levels were determined for lean **(A)**, obese **(B)**, and T2DM **(C)** subjects. **(D)** VLDL-cholesterol levels were higher in obese and T2DM subjects compared to lean subjects and the HIIT reduced VLDL-cholesterol in T2DM subjects. **(E)** LDL-cholesterol levels were higher in obese and T2DM subjects, which was reduced by the HIIT. **(F)** HDL-cholesterol levels were higher in obese subjects than in lean subjects, and were increased by the HIIT only in lean subjects. **(G)** The total plasma cholesterol to HDL-cholesterol (TC/HDL-C) ratios were significantly higher in obese and further higher in T2DM than in lean group and were in a trend of reduction by the HIIT in T2DM subjects. Statistical analysis were performed with 2-way ANOVA with Tukey’s multiply comparison analysis *p < 0.05; **p < 0.01; ***p < 0.001.

**FIGURE 3:**
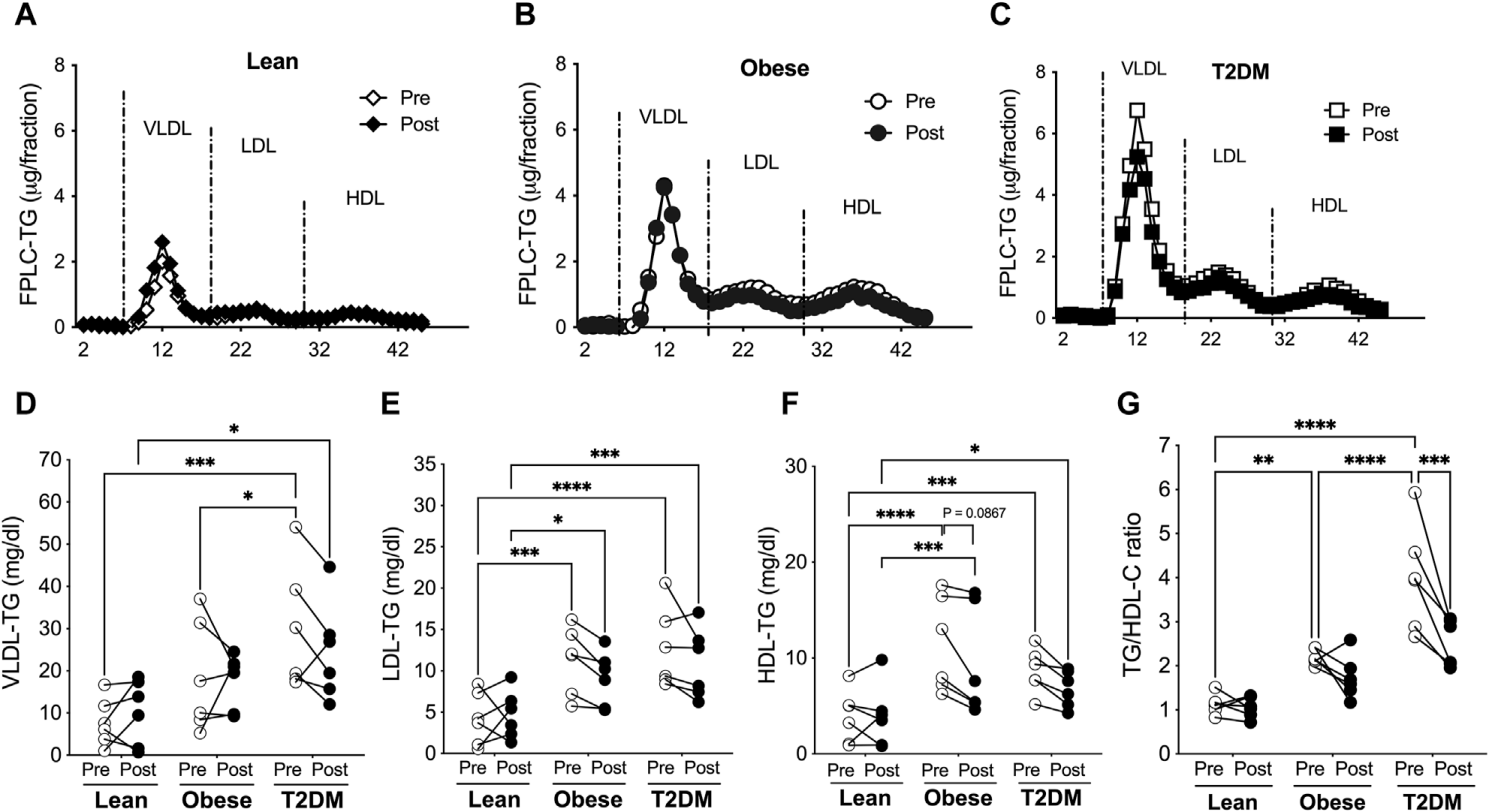
VLDL-, LDL-, and HDL-TG contents were higher in obese and T2DM subjects than in lean subjects, and the short-term HIIT reduced LDL- and HDL-TGs only in obese subjects. Blood lipoproteins were saperated with FPLC and lipoprotein TGs were determined for lean **(A)**, obese **(B)**, and T2DM **(C)** subjects. **(D)** VLDL-TG contens were higher in T2DM subjects than in lean and obese subjects before the HIIT program, and stayed higher in T2DM than in lean subjects after the HIIT program. **(E)** LDL-TG levels were higher in obese and T2DM individuals than in lean subjects before and after the HIIT program. **(F)** HDL-TG levels were higher in obese and T2DM subjects than in lean subjects before and after the HIIT program, and there was a trend of reduction in HDL-TG only in obese subjects by the HIIT. **(G)** The total plasma TG to HDL-cholesterol (TG/HDL-C) ratios were significantly higher in obese and further higher in T2DM than in lean subjects and were reduced by the HIIT program only in T2DM subjects. Statistical analysis were performed with 2- way ANOVA with Tukey’s multiply comparison analysis *p < 0.05; **p < 0.01; ***p < 0.001; ****p < 0.0001.

Plasma lipoproteins were separated with fast performance liquid chromatography (FPLC), and lipoprotein-cholesterol and -TG contents were determined for lean, obese, and T2DM subjects (Figures 2A–C). VLDL-cholesterol (VLDL-C) concentrations were higher in obese and T2DM subjects than in lean subjects; the short-term HIIT reduced VLDL-C only in T2DM subjects (Figure 2D). The average LDL-C content was about 2-fold higher in obese and T2DM subjects than in lean subjects; the HIIT significantly reduced LDL-C in obese and T2DM subjects (Figure 2E). HDL-C content was slightly but significantly higher in obese than in lean subjects; the HIIT increased HDL-C only in lean subjects (Figure 2F). While hypercholesterolemia is atherogenic, HDL-C has been considered “good cholesterol” regarding CAD. The ratio of total cholesterol to HDL-C (TC/HDL-C) has been used to predict future CAD events in the clinic, and a ratio number higher than four is considered a high risk for heart disease (Lemieux et al, 2001). The ratio of TC/HDL-C was 4.079 ± 0.344 and 4.952 ± 0.339 in obese and T2DM subjects, respectively, which was higher than in lean subjects (3.014 ± 0.169, Figure 3G). The HIIT did not significantly reduce HC/HDL-C ratios in any group except for a trend of decrease in the T2DM group (*p* = 0.0658, Figure 3G).

The distribution of TG in lipoproteins was similar to the distribution of cholesterol in lipoproteins. VLDL-TG concentrations were not different between lean and obese subjects before and after the HIIT program (Figure 3). VLDL-TG concentrations were higher in T2DM than in lean and obese subjects before the HIIT and were maintained at higher levels compared to lean subjects after the exercise training (Figure 3D). LDL- and HDL-TG levels were higher in obese and T2DM than in lean subjects before and after the HIIT program (Figure 3). The short-term HIIT did not significantly change TG distribution in lipoproteins in any group except for a trend of decrease in HDL-TG in obese subjects (Figures 3A–F). While TC/HDL-C ratios are used to predict CAD risk, TG/HDL-C ratios are considered indicators for metabolic disorders and insulin resistance (Iwani et al, 2017; Liu et al, 2022). TG/HDL-C ratios range between 0.5–1.9 for healthy people, and ratios above 3.0 indicate significant insulin resistance and CAD risk (Iwani et al., 2017; Liu et al., 2022). The TG/HDL-C ratios were higher in obese than in lean subjects (2.197 ± 0.073 vs. 1.124 ± 0.092, *p* < 0.01) and were further increased in T2DM subjects compared to obese subjects (3.995 ± 0.486 vs. 2.197 ± 0.073, *p* < 0.0001, Figure 3G). The HIIT significantly reduced TG/HDL-C ratios only in T2DM subjects to 2.512 ± 0.218 (*p* < 0.001, Figure 3G).

### 3.3 The HIIT program improved the antioxidant capacity of HDL in all subjects and increased cholesterol efflux capacity in lean subjects

While the short-term HIIT program improved many aspects of lipid profile in obese and T2DM subjects, HDL-C levels were unaffected. Given that HDL function is essential for delaying CAD, we next performed functional assays with HDL particles isolated from the plasma samples before and after the HIIT program.

**FIGURE 4:**
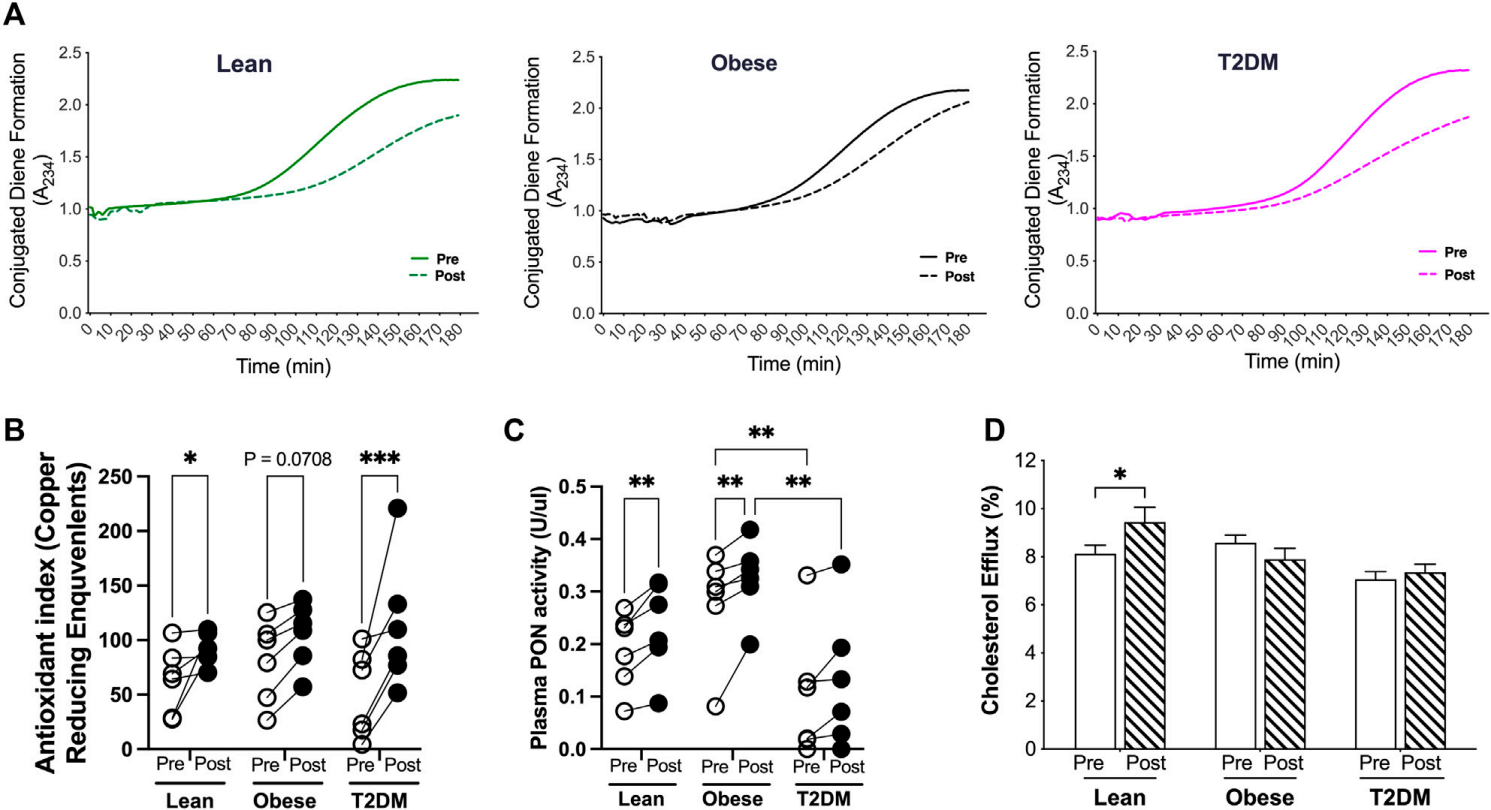
Antioxidant and cholesterol efflux capacities of HDL were changed by the HIIT in lean, obese, and T2DM subjects. ApoB-free serum of each sample was used for the function assays to exame the antioxidant and cholesterol efflux capacities as discribed in the Methods and Material section. **(A)** HDL particles isolated from post-HIIT plasma samples of all subjects impleded copper-induced LDL oxidation. **(B)** The capacity of reduction of copper II to copper I by HDL particles was inproved by the HIIT program in lean and T2DM subjects. **(C)** Plasma total paraoxonase (PON) activities were higher in obese than in T2DM subjects before and after the exercise training, and the HIIT program improved the PON activities in lean and obese subjects. **(D)** Cholesterol efflux capacity was improved by the HIIT only in lean subjects. Statistical analysis were performed with 2-way ANOVA with Tukey’s multiply comparison analysis *p < 0.05; **p < 0.01; ***p < 0.001.

The antioxidant capacity of HDL is important to suppress LDL oxidation and retention in the subendothelial space during atherosclerosis progression (Linton et al., 2023). To examine the antioxidant capacity of HDL, we first performed the kinetic assays for LDL oxidation in the presence of HDL particles isolated from plasma samples pre- and post-HIIT. HDL particles from post-HIIT plasma of all subjects delayed LDL oxidation by shifting the curves for the formation of conjugated diene, an oxidative product of LDL, toward the right (Figure 4A). These results indicate that the short-term HIIT improved the antioxidant capacity of HDL in all participants from lean, obese, and T2DM groups. To further quantify the antioxidant capacity, the reduction of copper oxidation was evaluated in the presence of HDL particles (Trachootham et al., 2008). The capacity of HDL to reduce copper oxidization was not significantly different between groups before the exercise training and was improved by the HIIT in lean and T2DM subjects (Figure 4B). Evidence from pre-clinical and clinical studies shows that the antioxidant capacity of HDL is largely accounted to the enzymatic activity of paraoxonase 1 (PON1) (Shokri et al, 2020). Plasma PON activities were examined, and the results showed that plasma PON activities were not different between lean and obese subjects but were higher in obese than in T2DM subjects before and after the exercise training (Figure 4C). The short-term HIIT increased PON activity in lean and obese subjects but not in T2DM participants (Figure 4C).

HDL particles accept cholesterol transported from the arterial wall and deliver cholesterol either back to the liver or to LDL particles in a process termed reverse cholesterol transport (RCT) (Linton et al., 2023). Impaired RCT is associated with increased CAD events (Zhu et al., 2018b). To examine the cholesterol efflux capacity, HDL particles from each sample before and after the HIIT program were added in the medium for culturing macrophages that were loaded with ^3^H-acylated-LDL. The cholesterol efflux capacity of HDL was presented as the percentage of ^3^H-radioactivity transported to HDL particles from macrophages (Zhu et al., 2018a; Zhu et al., 2018b). The cholesterol efflux capacity was not different between lean, obese, and T2D subjects before the exercise training, and was increased by the short-term HIIT program only in lean subjects (Figure 4D).

To better understand the improvement in HDL function by the HIIT program, we performed multiple logistic regression analysis for changes in HDL antioxidant capacity with the changes in other metabolic parameters (Table 1; Supplemental Figure 1). In addition to the inverse correlations with changes in atherogenic lipids, the improvement in HDL antioxidant capacity was inversely correlated with changes in HDL-TGs. Furthermore, changes in plasma PON activity were associated with changes in apoC2 and HDL-C but inversely correlated with changes in LDL-TG and HDL-TG (Supplemental Figure 1). These results together indicate that hyperlipidemia, especially the TG content in HDL particles, may have negative impact on HDL function by interrupting the enzyme activities of apolipoproteins.

**TABLE 1:** Metabolic factors whose changes significantly correlate with the change in HDL capacity for antioxidant function by multiple logistic regression analysis (more data shown in Supplemental Figure 1).

	ΔPON activity	ΔTC	ΔTG	ΔVLDL-TG	ΔLDL-TG	ΔHDL-TG	ΔVLDL-C	ΔTG/HDL-C
95% Confidence interval of R	−0.050 to 0.745	−0.846 to −0.227	−0.897 to −0.418	−0.812 to −0.121	−0.844 to −0.220	−0.799 to −0.084	−0.868 to −0.304	−0.832 to −0.179
Pearson R value	0.427	−0.627	−0.741	−0.557	−0.623	−0.530	−0.675	−0.596
*p*-value	0.77	0.005	<0.001	0.016	0.006	0.024	0.002	0.009

PON, paraoxonase; TC, total cholesterol; TG, triacylglycerol; VLDL-C, very low-density lipoprotein cholesterol; HDL-C, high density lipoproteins cholesterol.

## 4 Discussion

In this study, we report that a short-term HIIT program reversed plasma TG levels in obese subjects and partially reversed plasma TG and cholesterol levels in T2DM subjects. While TG contents in lipoproteins were not significantly changed, the HIIT program reduced VLDL-C and LDL-C concentrations in T2DM subjects and increased HDL-C concentrations in lean subjects. The improvement in lipid profile was in line with our previous report that the HIIT increased VO_2peak_ associated with increased insulin sensitivity in subjects from all three groups (Reyna et al., 2013). Furthermore, we show that the short-term HIIT program improved the antioxidant capacity of HDL in all subjects and increased the cholesterol efflux capacity in lean subjects. The impact of the HIIT on HDL functions is metabolic context dependent and associated with a reduction in atherogenic lipids in the plasma.

We report that the short-term HIIT program increased cholesterol efflux capacity of HDL and HDL-C in lean subjects. Studies have shown that changes in HDL-C concentrations by exercise training depend on subjects’ baseline lipid profiles, and training programs with extended periods are required for subjects with hyperlipidemia to increase HDL-C (Franczyk et al., 2023). Although HIIT is an efficient training for reducing hyperlipidemia, a 15-day continuous training may not be sufficient to improve HDL-C in obese and T2DM subjects. It is worth noticing that the clinical practice of HIIT for patients with dyslipidemia may need to be limited to once per week to minimize the symptoms related to overreaching (Gottschall et al, 2020). We also show that the increase in HDL-C in lean subjects was associated with increased cholesterol efflux capacity but disassociated with changes in circulating apoA1 or CETP amounts. This observation indicates that the short-term HIIT program modified additional regulatory steps in RCT to steps regulated by ApoA1 and CETP to increase HDL-C. In line with our observations, in a study with prolonged exercise training, HDL-C was modestly increased without any change in apoA1 protein amounts (Thompson et al, 1988). Furthermore, HDL-C concentrations are reported to be higher in trained subjects, and changes in HDL-C are positively correlated with increases in aerobic capacity by exercise training (Schwartz, 1988). We were unable to perform a correlation analysis between the changes in HDL-C and VO2peak due to the missing values for three subjects after the HIIT training. Christou et al (2024) reported that oxygen uptake at the first ventilatory threshold (VO2VT1) is more accurate than VO2peak for aerobic capacity in heart failure patients, and VO2VT1 should be considered in future studies for evaluating the impact of exercise training on HDL functions. The disassociation between HDL-C concentrations and apoA1 was also seen in obese subjects in the current study. HDL-C concentrations were higher in obese than lean subjects at the baseline but were disassociated with higher functional quality, such as the cholesterol efflux capacity. Notably, HDL-TG content was higher in the obese than in lean subjects at the baseline. Our analysis showed that reduction in HDL-TG was associated with improvements in atherogenic blood lipid concentrations and correlated with increases in HDL antioxidant capacity. Clinical studies have shown that HDL-TG content is positively associated with the risk of myocardial infarction (Lamarche et al, 1999; Holmes et al, 2018; Ito and Ito, 2020). Observations from our study and others’ indicate that TG enrichment in HDL may impair their function of HDL particles.

The short-term HIIT improved the antioxidant capacity of HDL in all subjects from different metabolic groups. Paraoxonase proteins (PON1 and PON3) in HDL particles play a key role in hydrolyzing and clearing lipid peroxides in circulation (Soran et al, 2015; Ruiz-Ramie et al, 2019). Increases in the antioxidant property of HDL in lean and obese subjects were associated with the increases in PON activities by the HIIT program. Other apolipoproteins, such as apoA1, apoA2, or CETP, are required for PON1 impeding lipid peroxide accumulation on LDL because highly purified PON1 proteins isolated from HDL could not reduce lipid peroxides (Draganov et al, 2005; Kontush and Chapman, 2010). These studies indicate that modification of other apolipoproteins by the HIIT contributed to the enhancement in the antioxidant capacity of HDL in T2DM subjects where PON activities were not significantly increased (Figures 4B, C). In agreement with our observation, evidence from independent studies has shown that exercise training promotes the antioxidant capacity of HDL in subjects of different metabolic contexts. Valimaki et al. (2016) reported that lipid peroxide clearing by HDL was elevated after an incremental maximal treadmill run to exhaustion. Roberts et al (2006) showed that a 21-day program of daily moderate-intensity exercise and dietary modification improved the antioxidant effect of HDL and reduced lipid peroxides in subjects with obesity and metabolic syndrome. Furthermore, a 3-month moderate-intensity exercise training program increased the HDL subfractions’ oxidative capacity and PON1 activity without significant changes in HDL-C (Casella-Filho et al, 2011). In another study with obese subjects, a 12-week exercise training program improved PON activities without affecting HDL-C content or cholesterol efflux capacity (Woudberg et al, 2018).

There were several limitations in our study. The sample size was small, especially for obese subjects whose metabolic regulations may span a wide range. A bigger sample size would better show metabolic significance between groups. In addition, lecithin cholesterol acyl transferase (LCAT) esterifies cholesterol and facilitates the transport of cholesterol esters to HDL particles, and phospholipid transport proteins (PLTP) are also involved in the regulation of HDL-C concentrations (Rousset et al, 2011). It would be interesting to investigate the regulation of LCAT and PLTP activities by exercise training in lean, obese, and T2DM subjects in future studies. Regarding HDL function, we could not examine the anti-inflammatory capacity of HDL with *in vitro* functional assays due to the limited amounts of plasma samples. We speculate that the HIIT improved the HDL capacity to protect endothelial cell injury from oxidized LDL, at least in lean and T2DM subjects, given that the ability of HDL particles to reduce the oxidized copper was improved in those subjects (Figure 4B).

In summary, our results show that a short-term HIIT program is sufficient to improve hyperlipidemia and HDL capacity of antioxidant function in obese and T2DM subjects, which might not be associated with increases in HDL-C concentrations. In addition to enhancing the antioxidant capacity, a short-term HIIT program could increase HDL-C concentrations and cholesterol efflux function in subjects without metabolic disorders. Furthermore, our data indicate that the TG content in HDL particles may play a negative role in the anti-atherogenic function of HDL.

## Data Availability

Publicly available datasets were analyzed in this study. This data can be found here: https://www.hindawi.com/journals/jdr/2013/107805/.
