# Supplementary figures and images for: Short-term HIIT impacts HDL function differently in lean, obese, and diabetic subjects
Source: Front Physiol. 2024 Aug 21;15:1423989. doi: 10.3389/fphys.2024.1423989 (PMC11371628; doi:10.3389/fphys.2024.1423989)

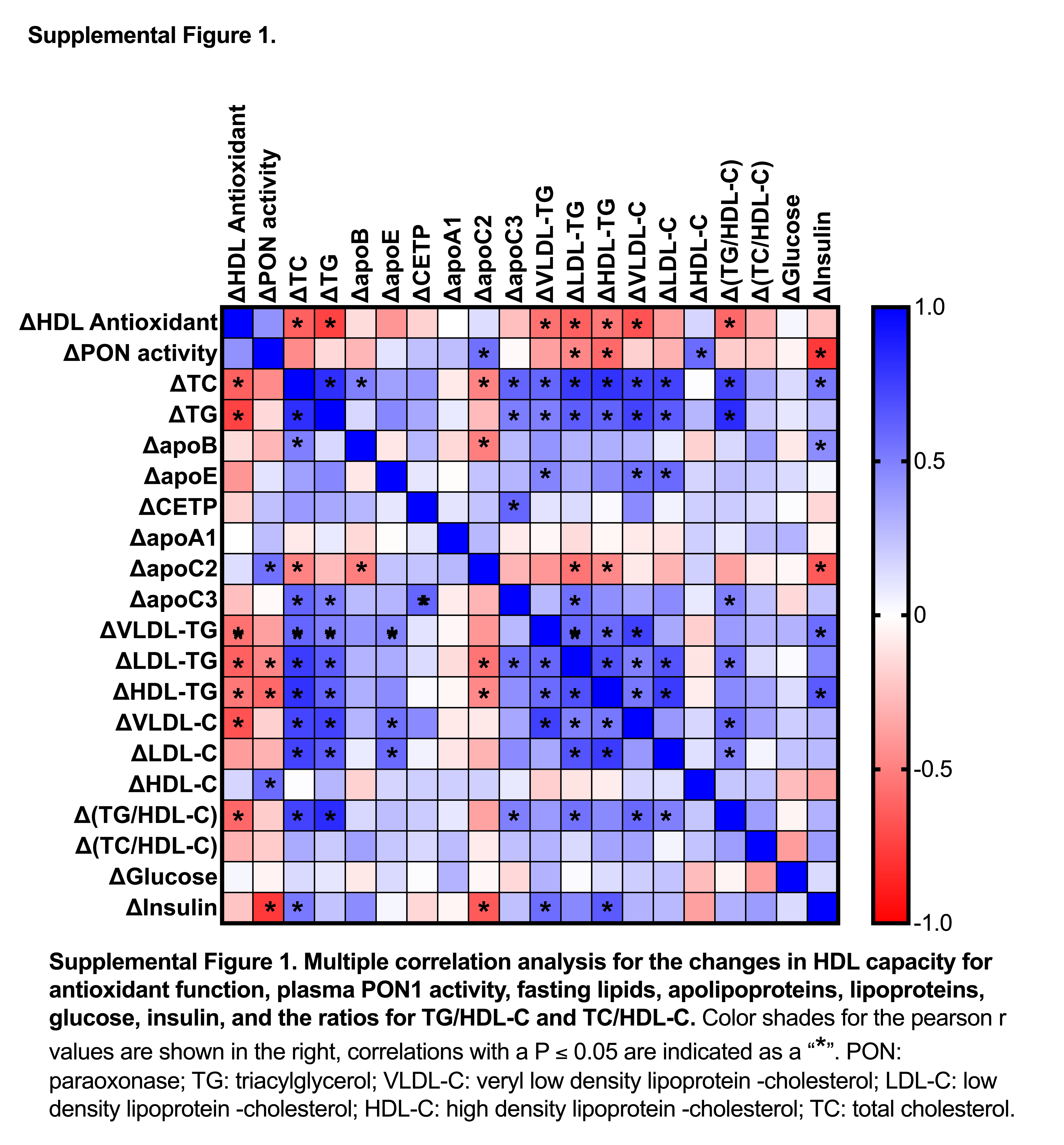

Supplement: Supplementary file 1 [file Image1.TIFF]
