# Supplementary material for: Short-term HIIT impacts HDL function differently in lean, obese, and diabetic subjects
Source: Front Physiol. 2024 Aug 21;15:1423989. doi: 10.3389/fphys.2024.1423989 (PMC11371628; doi:10.3389/fphys.2024.1423989)
Supplement: Supplementary file 2 [file Table1.docx]

**Supplemental Table 1:** Subject characteristics, metabolic parameters, and inflammatory markers pre and post exercise training.

|  | Lean | | Obese | | T2DM | |
| --- | --- | --- | --- | --- | --- | --- |
|  | (n=6) | | (n=6) | | (n=6) | |
|  | Pre | Post | Pre | Post | Pre | Post |
| **Subject characteristics** | | | | | | |
| Age (years) | 39.3±2.1 | ⎯ | 41.7±3.9 | ⎯ | 50.5±4.9 | ⎯ |
| Gender (F/M) | 5/1 | ⎯ | 5/1 | ⎯ | 5/1 | ⎯ |
| Weight (kg) | 65.0±4.0 | 64.4±3.9 | 81.7±3.8^*^ | 82.2±3.7^#^ | 85.8±2.7^*,^^ | 86.2±2.3^#,Ψ^ |
| BMI (kg/m2) | 24.8±1.1 | 24.6±1.1 | 30.4±1.2^*^ | 30.5±1.2^#^ | 34.6±0.9^*,^^ | 34.7±0.7^#,Ψ^ |
| **Metabolic parameters** | | | | | | |
| NEFA AUC during  OGTT (μmol/L•2h) | 26.1±2.7 | ⎯ | 37.3±6.2 | ⎯ | 62.0±7.7^*,^^ | ⎯ |
| NEFA during OGTT (μmol/L) | 0.42±0.05 | ⎯ | 0.57±0.09 | ⎯ | 0.78±0.03^*^ | ⎯ |
| HbA1c (%) | 5.2±0.1 | ⎯ | 4.9±0.1 | ⎯ | 7.4±0.8^*,^^ | ⎯ |
| Fasting insulin (μg/mL) | 2.4±0.9 | 2.4±0.7 | 17.8±11.5^*^ | 15.1±5.7^4^ | 9.7±1.4^5^ | 11.2±1.9^#^ |
| Fasting glucose (μg/mL) | 90.2±1.4 | 89.2±2.4 | 94.1±1.8 | 95.6±1.4 | 128.1±20.9^*, ^^ | 139.5±24.7^#,Ψ^ |
| Fasting Cho (mg/dL) | 89.6 ± 9.6 | 85.1 ± 6.8 | 146.7 ±11.2^*^ | 118.9 ±14.5^#^ | 163.9 ± 5.4^*^ | 122.0±10.6^#,&^ |
| Fasting TG (mg/dL) | 34.2 ± 5.3 | 37.9 ± 4.2 | 79.8 ± 4.6^*^ | 54.0 ± 6.7^&^ | 134 ± 24.3^*,^^ | 74.2±12.7^#,&^ |
| **Inflammatory markers** | | | | | | |
| hs-CRP (μg/mL) | 1.71±0.6 | 3.57±1.3 | 6.02±3.9 | 4.87±1.9 | 4.65±1.2 | 4.37±1.2 |
| Endothelin-1 (pg/mL) | 2.27±0.3 | 2.92±0.8 | 2.98±0.3 | 2.58±0.3 | 5.23±0.1^*,^^ | 5.27±0.7^#,Ψ^ |
| sICAM-1 (ng/mL) | 212±35 | 191±13 | 172±20 | 179±18 | 289±16^*,^^ | 277±19^#,Ψ^ |
| **VO2peak** | 18.3±2.1 | 22.9±2.4^7^ | 16.8±0.6 | 18.0±2.0^7^ | 13.3±1.1^*^ | 14.3±1.6^#,7^ |

Data are mean ± SE, statistical analysis was performed with one-way or two-way ANOVA with Tukey’s multiply comparison analysis. P<0.05; ^*^: versus Lean/Pre; ^#^: versus Lean/Post; ^^^: versus Obese/Pre; ^Ψ^: versus Obese/Post; ^&^: Pre vs. Post within the same metabolic group. ^4^: P=0.0629; ^5^: P=0.0551; P<0.01 ^7^: P=0.0473, two-way ANOVA with HIIT as main effect model. Pre: pre-exercise; Post: post-exercise; BMI: body mass index; NEFA: non-esterified fatty acids; AUC: area under curve; OGTT: oral glucose tolerance test; HbA1c: Hemoglobin A1c; Cho: cholesterol; TG: triacylglycerol; hs-CRP: high-sensitivity C-reactive protein; sICAM-1: soluble intercellular adhesion molecule-1.
